# Supplementary material for: Comprehensive Annotation and Functional Exploration of MicroRNAs in Lettuce
Source: Front Plant Sci. 2021 Dec 24;12:781836. doi: 10.3389/fpls.2021.781836 (PMC8739914; doi:10.3389/fpls.2021.781836)
Supplement: Supplementary file 3 [file Data_Sheet_9.ZIP › GSM2230752.plot/Lsa-miRN1652a_Lsat_1_v5_gn_1_93941.1_125_TPlot.pdf]

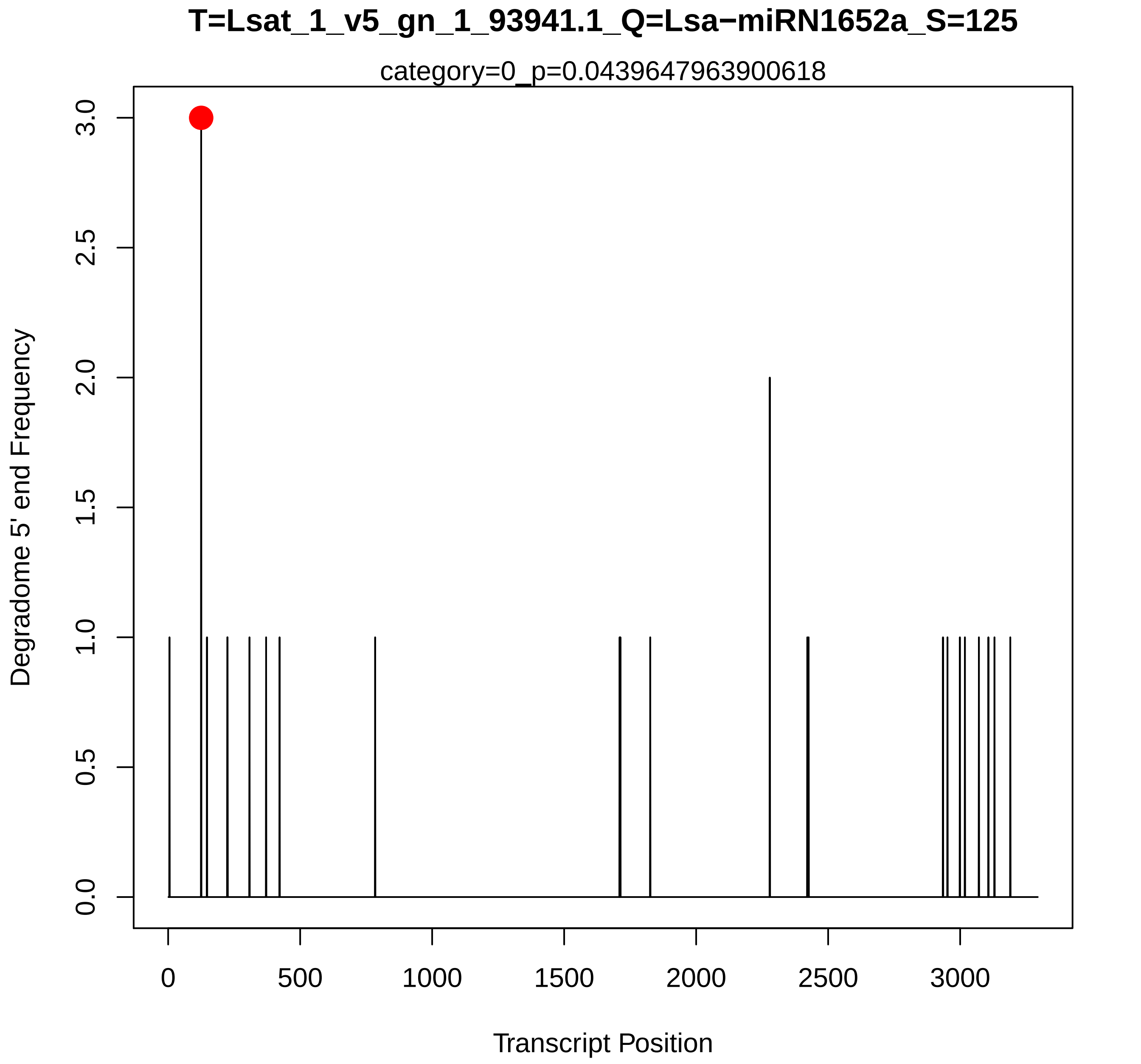
T=Lsat_1_v5_gn_1_93941.1_Q=Lsa−miRN1652a_S=125
category=0_p=0.0439647963900618
Degradome 5' end Frequency
0.0
0.5
1.0
1.5
2.0
2.5
3.0
0
500
1000
1500
2000
2500
3000
Transcript Position
